# Use of the Desmopressin Clamp in Hyponatremia: Smooth Sailing or Turbulent Waters?

**DOI:** 10.34067/KID.0000000953

**Published:** 2025-09-25

**Authors:** Aruna Phekoo, Matthew A. Sparks

**Affiliations:** 1 Division of Nephrology, Department of Medicine, Duke University School of Medicine, Durham, North Carolina; 2 Durham VA Health Care System, Durham, North Carolina

**Keywords:** hyponatremia

Since the first report of osmotic demyelination syndrome (ODS) by Adams and Victor in 1959,^1^ the safest approach to correcting hyponatremia has remained a topic of ongoing debate. Although ODS is most commonly associated with rapid correction of chronic hyponatremia, it has also been reported in other contexts, such as the rapid correction of hyperglycemia, traumatic brain injury, and refeeding syndrome. Although ODS is a rare complication, its devastating neurologic consequences have led to a longstanding emphasis on cautious and controlled sodium correction to minimize the risk of iatrogenic harm.

Recently, however, this traditional approach has come under scrutiny. In 2023, Macmillian *et al.*^2^ published a multicenter cohort study with 22,858 patients with hyponatremia and evaluated the incidence of ODS. Rapid correction of serum sodium occurred in 17.7% of patients in the study. Twelve patients developed ODS (0.05%), but seven did not have rapid correction of sodium. Interestingly, five or fewer patients whose initial sodium level was >125 mmol/L developed ODS without overcorrection but notably had hypernatremia after the correction of hyponatremia and known risk factors for ODS. Another multicenter observational study by Seethapathy *et al.*^3^ involving 3274 patients with sodium <120 mmol/L found that slower correction was associated with higher mortality and longer hospital stays. Among the seven patients who developed ODS, five had not exceeded an 8 mmol/L correction in 24 hours—challenging the notion that correction threshold is the most important risk factor for ODS. Similarly, a meta-analysis by Ayus *et al.*^4^ including 16 cohort studies (*n*=11,811) showed that rapid sodium correction was associated with lower mortality and shorter hospitalizations, without a significant increase in ODS risk. These findings have sparked debate over whether the conventional practice of slow correction is always appropriate. Although these studies offer compelling evidence that the risk of ODS with overcorrection of hyponatremia may be overstated, these studies could not completely adjust for confounders and did not account for the causes or chronicity of hyponatremia. Regardless of the exact risk, ODS remains a real and preventable consequence of overcorrection of sodium in high-risk patients. Clinicians must carefully manage hyponatremia to minimize patient harm.

Desmopressin (1-desamino-8-D-arginine vasopressin, DDAVP) is a synthetic analog of vasopressin developed by Ferring Pharmaceuticals in the 1960s and marketed for the treatment of bleeding disorders and central diabetes insipidus. Engineered to retain vasopressin's antidiuretic effects while minimizing vasopressor activity, DDAVP has a longer t_1/2_ and is resistant to degradation by vasopressinases, making it effective in gestational diabetes insipidus where placental enzymes degrade endogenous vasopressin.

More recently, DDAVP has emerged as a therapeutic adjunct in the management of severe hyponatremia. By binding to vasopressin V2 receptors in the renal collecting ducts, it enhances free water reabsorption, thereby slowing rapid rises in serum sodium. Colloquially known as the “DDAVP clamp,” the concurrent administration of DDAVP with a calculated dose of hypertonic saline enables precise control of sodium correction. This approach limits renal-free water clearance and prevents the abrupt drop in antidiuretic hormone that can trigger rapid water diuresis and overcorrection.

Although the DDAVP clamp may allow for a more predicted rate of rise of sodium, concerns persist around delayed sodium normalization, fluid retention, and prolonged hospitalization. There is a paucity of data to guide the use of the DDAVP clamp, and there is no established consensus regarding the optimal timing, dosing, or patient selection for this approach. In the absence of large randomized trials, practice patterns are highly variable across institutions.

Stern *et al.,*^5^ in their review of the management of hyponatremia in the intensive care unit, proposed giving DDAVP proactively with hypertonic saline to prevent overcorrection. Subsequent studies conducted by Sood *et al.*^6^ and Macmillan *et al.*^7^ showed a lower incidence of overcorrection with the use of proactive DDAVP and hypertonic saline concurrently; however, these studies were limited by small sample size and significant heterogeneity (variability in patient populations, DDAVP dosing regimens, timing of administration, and concurrent use of hypertonic saline), respectively.

In this issue of *Kidney360,* AlShanableh *et al*.^8^ conducted a retrospective cohort study over a 5-year period across four academic hospitals in Pennsylvania to compare outcomes in patients with severe hyponatremia (sodium ≤120 mmol/L) who were treated with proactive DDAVP with concomitant hypertonic saline versus those treated with hypertonic saline with the occasional rescue DDAVP. The cohort was divided into two groups: a proactive group, in which DDAVP was administered within 2 hours of initiating hypertonic saline, and a nonproactive group, which included patients who received reactive or rescue DDAVP or no DDAVP at all.

Patients were excluded if they had an eGFR <15 ml/min per 1.73 m^2^ or were on KRT, had prior use of DDAVP, a serum glucose >300 mg/dl, or missing sodium measurements at the 24-hour or 48-hour time points (defined as no sodium value within 12 hours of each). A total of 184 patients met inclusion criteria and were included in the study.

The authors classified the etiology of hyponatremia by reviewing clinical and laboratory data. Rates of sodium correction were estimated by subtracting the last sodium value before hypertonic saline initiation from the estimated 24-hour sodium. To estimate the change during the second 24 hours, they subtracted the estimated sodium level at 24 hours from the estimated level at 48 hours. Because sodium measurements were not uniformly collected at exactly 24 or 48 hours, the authors used a linear interpolation formula developed by Geoghegan *et al.*^9^ to estimate sodium values at those time points.

The primary outcome was overcorrection, defined as an increase in sodium of more than 8 mEql/L within the first 24 hours after initiation of hypertonic saline.

The proactive DDAVP group had significantly lower rates of overcorrection compared with the reactive group (6.8% versus 27.1%, *P* = 0.009). In addition, a greater proportion of patients in the proactive group achieved the target sodium correction of 4–6 mEq/L within the first 24 hours of therapy (34% versus 16%, *P* = 0.021). The mean sodium increase during the first 24 hours was 5.6 mEq/L in patients who did not receive DDAVP, 7.9 mEq/L in those who received rescue DDAVP, 6.8 mEq/L in the reactive DDAVP group, and 4.4 mEq/L in the proactive DDAVP group.

Sodium levels were monitored more frequently in the proactive cohort. There were no significant differences in adverse events between the groups. Importantly, no cases of ODS were reported in either cohort, although the study was not designed to detect such cases. There are several limitations to note for this study. First, the retrospective nature of this study introduces the potential for selective bias and confounding. Because treatment decisions were made at the discretion of individual clinicians rather than by protocol, it is possible that patients who received proactive DDAVP were managed more cautiously overall, supported by the higher frequency of sodium checks in this group, contributing to lower rates of overcorrection. In addition, clinical variables such as fluid balance, timing of potassium repletion, and concurrent medications may not have been consistently recorded, limiting the ability to fully adjust for confounders. The reliance on estimated rather than precise 24-hour and 48-hour sodium levels, along with incomplete correction for hyperglycemia due to missing glucose data, also has the potential to introduce measurement bias.

Despite these limitations, there are several notable strengths. This is the largest retrospective analysis to date on the use of proactive DDAVP with hypertonic saline in severe hyponatremia. In addition, the study focused on a high risk population, only including patients with sodium<120 mEq/L, and all patients in the study received hypertonic saline. Previous retrospective studies evaluating the use of proactive DDAVP were small and the definition of “proactive” use of DDAVP varied. A recent randomized trial conducted by Paktochanon *et al.,*^10^ which included 49 patients with a serum sodium of <125 mEq/L, investigated the safety and efficacy of proactive DDAVP with hypertonic saline versus reactive DDAVP in treating severe symptomatic hyponatremia. The primary outcome was the difference in incidence of overcorrection between proactive and reactive strategies. The study found no significant difference in the overcorrection incidence between the proactive and reactive groups; however, at 48 hours, the proactive group exhibited a higher but still safe change in sodium levels compared with the reactive group. This suggests that early DDAVP administration in the proactive group may have facilitated a faster improvement in sodium in a safe manner and did not lead to relowering of the sodium. Unfortunately, the small sample size of this study limits any conclusions we may draw and is subject to type II error and lack of generalizability.

In summary, the study by AlShanableh *et al.* adds to the growing evidence that proactive DDAVP, when used with hypertonic saline, may be an effective strategy for safely managing severe hyponatremia. Although limited by its retrospective design, the findings suggest that the “DDAVP clamp” can help reduce the risk of overcorrection in high-risk patients without increasing adverse outcomes.

Future prospective randomized controlled trials comparing proactive DDAVP with concomitant hypertonic saline with a more reactive approach to overcorrection are essential to confirm these findings and to clarify their effect on overcorrection, the incidence of ODS, mortality, and length of hospitalization. Until then, the “DDAVP clamp” remains a valuable tool that can help clinicians in the complex management of hyponatremia.
